# Heated Corn Oil and 2,4-Decadienal Suppress Gastric Emptying and Energy Intake in Humans

**DOI:** 10.3390/nu13041304

**Published:** 2021-04-15

**Authors:** Hideaki Kashima, Ayumi Honma, Saori Kamimura, Saki Nishimura, Takashi Sano, Shoji Matsumoto, Masako Yamaoka Endo, Yoshiyuki Fukuba

**Affiliations:** 1 School of Health Sciences, Prefectural University of Hiroshima, 1-1-71 Ujina-higashi, Minami-ku, Hiroshima 734-8558, Japan; a.honma.pu.hiroshima@gmail.com (A.H.); s.kamimura.pu.hiroshima@gmail.com (S.K.); yamaoka@pu-hiroshima.ac.jp (M.Y.E.); fukuba@pu-hiroshima.ac.jp (Y.F.); 2 J-Oil Mills, Inc., 7-41 Oogurocho, Tsurumi-ku, Yokohama 230-0053, Japan; saki.nishimura@j-oil.com (S.N.); takashi.sano@j-oil.com (T.S.); shoji.matsumoto@j-oil.com (S.M.)

**Keywords:** gastric emptying rate, food intake, energy intake, oil, 2,4-decadienal, appetite, ultrasonography, heating, ^13^C breath test

## Abstract

Consumption of 2,4-decadienal (2,4-DD) delays gastric emptying (GE) rate in animals. Oil heating produces 2,4-DD and other aldehydes. Here we examined whether heated oil affects GE rate and food intake in humans, and whether it is mediated by 2,4-DD. In the first experiment, 10 healthy volunteers consumed 240-g pumpkin soup with 9.2 g of heated (HO) or non-heated corn oil (CO). Subsequently, 17 participants consumed pumpkin soup containing 3.1 g of either heated corn oil (HO), 1 mg 2,4-DD + non-heated corn oil (2,4-DD), or non-heated corn oil (CO). Sixty minutes following pumpkin soup, cod roe spaghetti was provided, and then energy intake was determined. To evaluate GE rate, ^13^C breath test (Experiment 1) and ultrasonography (Experiments 1 and 2) were used. The results from the Experiment 1 confirmed that consumption of heated corn oil reduced GE rate. Experiment 2 showed a delayed GE rate in HO and 2,4-DD trials compared with CO trial (*p* < 0.05). Energy intake was approximately 600–650 kJ lower in HO and 2,4-DD trials compared with CO trial (*p* < 0.05). These findings suggest that 2,4-DD, either formed by oil heating or added to food, contributes to suppressing GE rate and energy intake.

## 1. Introduction

The gastrointestinal (GI) tract plays an important role not only in digestion and absorption of ingested food, but also in determining appetite, energy intake, and postprandial glycemic excursions [1,2,3]. Through gastric emptying (GE) the stomach controls the rate of delivery of ingested food to the small intestine to optimize digestion and absorption. When nutrients enter the small intestine, they generate feedback signals that slow GE rate and suppress appetite through neural mechanisms as well as hormonal mechanisms (secretion of cholecystokinin [CCK] and glucagon-like peptide, and suppression of ghrelin) [4]. CCK is a hormone produced by secretory I-cells of the upper part of the digestive tract, and has various physiological functions such as increase in pancreatic enzyme secretion, suppression of GE rate, and induction of satiety [5]. If secretion of CCK can be increased or decreased depending on the content of a meal, GE rate may be altered, resulting in modulation of food intake and glycemic response [4,5].

Besides meal composition and size, the sequence of macronutrient consumption during a meal has recently been recognized as an important regulator of postprandial glycemia and appetite control via modulation of GE rate [6]. Accumulated evidence suggests that pre-meal consumption of protein and fat preloads can markedly reduce postprandial hyperglycemia and increase satiety via delaying GE rate and enhancing glucose-stimulated insulin release associated with increasing gut-derived hormones [7,8,9]. Meal sequence (consumption of e.g., meat, fish, or vegetables before rice) can play a role in postprandial glucose control through delaying GE rate and enhancing incretin secretion [10,11,12]. Such nutritional strategies without pharmacological treatments may be a simple, effective, and safe tool for prevention and management of postprandial hyperglycemia and overeating. Given that adherence to lifelong nutritional interventions involving energy restriction is often poor, there is a need for further alternative dietary strategies.

Beside the meal content and sequence, GE rate might be also influenced by way of cooking (e.g., heating oil). Benini et al. [13] reported that consumption of foods heated for 12 min with 10 g of olive oil reduced GE rate as assessed by ultrasonography, compared with consuming the same food but with added fresh, i.e., non-heated olive oil. However, it is insufficiently clear which of the various aldehydes formed during oil heating are responsible for modulating the GE rate. Candidates for aldehydes that can inhibit GE rate have been identified by Nakajima et al. [14], including a typical degradation product “2,4-decadienal (2,4-DD)” of linoleic acid, a fried food flavor [14]. In addition, a recent study investigated the effect of 2,4-DD on GE rate in rats [15], and found that the GE rate was significantly inhibited after consuming 2,4-DD-containing food compared with food without 2,4-DD [15]. However, it is unknown whether 2,4-DD can also inhibit GE rate in humans.

2,4-DD is formed during heating intact oil [16]. Thus, previous reports in humans showing inhibition of GE rate induced by oil heating [13] may be partially explained by the presence of 2,4-DD. However, when intact oils are heated, various aldehydes are formed besides 2,4-DD [14,16]. Therefore, it is unclear whether 2,4-DD formed by heating oil plays a role in suppressing GE rate in humans like it does in rats [15]. Moreover, when GE rate can be inhibited by consumption of 2,4-DD or heated oil, differences in subsequent food intake could be expected, but this has not been investigated. Therefore, the purpose of our study was to examine whether GE rate and food intake are reduced by 2,4-DD or by other components of the heated oil. To distinguish the effects of 2,4-DD, we examined how heated corn oil (containing 2,4-DD and other aldehydes formed during heating), intact corn oil + 2,4-DD (containing 2,4-DD, without other aldehydes normally produced during heating), and non-heated oil (no 2,4-DD and no aldehydes) affect the GE rate and food intake.

## 2. Materials and Methods

### 2.1. Participants

The study protocol fully complied with the Declaration of Helsinki, and the study was approved by the Prefectural University of Hiroshima Ethics Committee (approval number: 17HH001). Each participant provided a written informed consent to participate before the study commencement. In the Experiment 1, 10 healthy young participants (one man and nine women; age: 22 ± 1 years; height: 163 ± 8 cm; weight: 56 ± 7 kg; body mass index [BMI]: 21 ± 2 kg/m^2^) were included. The Experiment 2 involved 17 healthy young participants (2 men and 15 women; mean age: 21 ± 1 years; mean height: 159 ± 7 cm; mean weight: 54 ± 5 kg; mean BMI: 21 ± 2 kg/m^2^). In the Experiment 1 (but not Experiment 2), female participants participated in both trials during the same phase of their menstrual cycles, because menstruation modulates insulin, blood glucose, GE rate, and glucagon-like peptide-1 concentrations [17]. In the Experiment 1, a man and women participated maximum once per week and month, respectively. In the Experiment 2, all participants participated maximum once per 3 days. The participants had no gastrointestinal symptoms, food allergies, or history of significant diseases such as cardiovascular disease (hypertension with average systolic blood pressure of 140 mm Hg or higher, or average diastolic blood pressure of 90 mm Hg or higher), and they were not taking any medications. The day before the experiment, the participants consumed a standardized meal (hashed rice) of 1962 kJ (Ginza hayashi, Meiji, Tokyo, Japan; Satounogohan, Satosyokuhin, Niigata, Japan) at 19:30–20:00 or 20:30–21:00. They arrived in the laboratory at 08:30 a.m. or 09:30 a.m. the following day. They abstained from strenuous exercise and consumption of alcohol or caffeine for at least one day before visiting the laboratory. According to a previous study that examined the effect of heated oil on GE rate, the sample size was calculated in the Experiment 1 [13]. Specifically, we compared gastric emptying index (i.e., total emptying time) between two groups, nonfried (control trial) and fried meal, using G*Power software (version 3.1.9.2). We obtained an effect size of 1.461 for a critical t of 2.570 and actual power of 0.813 at α of 0.05 and power (1 − β) of 0.80; based on that, the calculated total sample size was six. In Experiment 2, in a pilot experiment (five participants), we compared food energy intake during trials of Con and HO or 2,4-DD. The mean values and standard deviation of food energy intake for Con, HO, and 2,4-DD were 6591 ± 1410 kJ, 5552 ± 1538 kJ, and 5909 ± 1608 kJ, respectively. Then, we ran a statistical power analysis based on this outcome using G*Power (version 3.1.9.2) with five participants per trial. We obtained an effect size of 1.063 for a critical F of 3.885 at α error probability of 0.05 and power (1 − β) of 0.80. The calculated total sample size was fifteen and, therefore, we planned to recruit a total of 17 participants to expect dropouts.

### 2.2. Preparation of Test Meal and Oil

The test meal (pumpkin soup) was cooked according to the following recipe. First, the 500 g of pumpkin was heated in the microwave, and the skin of pumpkin was peeled. Then, pumpkin and 300 g of onion were cut into small pieces and boiled in a pan with consommé soup for 40 min. Third, pumpkin and onion were then homogenized with a blender. Subsequently, 300 g of milk was added, and then water (approximately 600 mL) was added to adjust the viscosity to 100 (mPa·S) or less as measured using viscometer. Finally, 240 g of pumpkin soup was included in a retort pouch and restored in a freezer at −20 °C until the day before the experiment. On the day of the experiment, the pumpkin soup was heated to 42–45 °C using microwave. In all experiments, the pumpkin soup was served at 39–43 °C. 2,4-Decadienal (2,4-DD) was formed by heating an intact corn oil (J-Oil Mills INC., Tokyo, Japan) in Experiment 1; specifically, 300 g of intact corn oil was heated in an oil bath at 160 °C and at 250 rpm for 60 min with stirring. In Experiment 2, 100 g of intact corn oil was heated in an oil bath at 160 °C and 450 rpm for 60 min with stirring, and refluxing at −1 °C. The amount of 2,4-DD consumed in this study was similar to that consumed in a single meal of fried foods (e.g., pan-fried potatoes [18]).

### 2.3. Study Protocol

**Figure 1:**
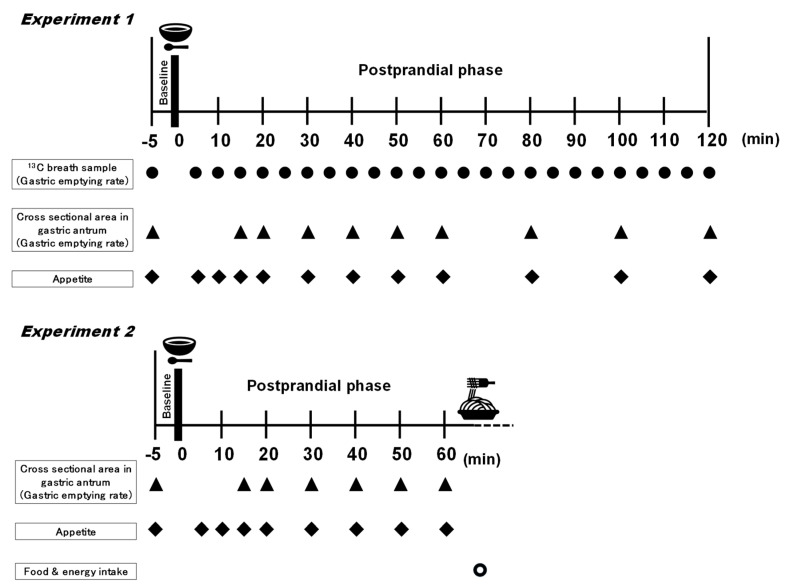
Scheme of the protocol of the study. In Experiment 1, following fasting baseline measurements for 5 min, the subjects were instructed to consume a 240 g pumpkin soup containing 9.2 g either intact corn oil (CO trial) or heated corn oil (HO trial) for 1–2 min, and then rested for 120 min. In Experiment 2, the subjects were instructed to consume a 240-g pumpkin soup containing 3.1 g of either non-heated corn oil (CO trial), heated corn oil (HO trial), or intact corn oil with added 1 mg 2,4-DD (2,4-DD trial) for 1–2 min, and then rested for 60 min. At 60 min after the consumption of pumpkin soup, cod roe spaghetti was given, and the participants were instructed to eat as much as they like until satisfied.

In Experiments 1 and 2, participants sat in a chair in a semi-supine position for 30 min in a quiet room in which the temperature and humidity were maintained at 24 ± 1 °C and 29 ± 5%, respectively. In Experiment 1, following fasting baseline measurements for 5 min, the subjects were instructed to consume a 240-g pumpkin soup containing 9.2 g either intact corn oil (CO) or heated corn oil (HO) for 1–2 min, and then rested for 120 min (Figure 1). In Experiment 2, following fasting baseline measurements for 5 min, the participants were instructed to consume a 240-g pumpkin soup containing 3.1 g of either non-heated corn oil (CO trial), heated corn oil (HO trial), or intact corn oil with added 1 mg 2,4-DD (2,4-DD trial) for 1–2 min, and then rested for 60 min. At 60 min after the consumption of pumpkin soup, cod roe spaghetti (13.9% protein, 17.8% fat, and 68.3% carbohydrate) was provided, and the subjects were instructed to eat as much as they like until satisfied. The spaghetti noodles (thickness of 1.6 mm) and cod roe sauce used were by Ma·Ma Macaroni Co., Ltd., Tochigi, Japan and Kewpie Co., Ltd., Tokyo, Japan, respectively. In order to exclude the possibility that the amount of food eaten depended on its palatability, prior to the study we asked all subjects which foods they liked, and based on that we selected cod roe spaghetti. The study had a randomized crossover design, and all participants came to the laboratory on two or three occasions in study 1 and 2, respectively. Namely, at first trial in each experiment, participants were alternatively allocated to groups, specifically to the CO (*n* = 5) and HO (*n* = 5) in the Experiment 1, and then, CO (*n* = 6), HO (*n =* 6), and 2,4-DD (*n =* 5) in the Experiment 2.

### 2.4. Measurements

#### 2.4.1. 2,4-DD Analysis

The amount of 2,4-DD, 2-octenal, and 2-heptenal of each sample oil were calculated in the following manner according to the method of previous study [18,19]. Namely, 500 mg of the sample oils were accurately weighed on a precision balance, and 100 μL of 11.0 mg/mL 2,6-dimethyl-5-heptenal in acetonitorile as an internal standard and 1 mL of acetonitorile were added. The extracts were vortexed for 30 min, separated into two layers by a centrifuge (3500 rpm, 10 min), and the upper layer was prepared as a gas chromatography/mass spectrometry (GC/MS) sample. GC/MS analysis was performed using an Agilent 7890A gas chromatograph (Santa Clara, CA, USA) equipped with a DB-WAXUI GC column (60 m, 0.25 mm inner diameter, 0.25 μm film thickness, Agilent, Santa Clara, CA, USA) and coupled with an Agilent 5975C MS detector (Santa Clara, CA, USA). The calibration curve was prepared using standard reagents for 2,4-DD (FUJIFILM Wako Pure Chemical Corporation, Osaka, Japan), 2-octenal and 2-heptenal (Tokyo Chemical Industry, Tokyo, Japan), and the concentration (ppm) was calculated based on the internal standard and the mass used for the analysis.

#### 2.4.2. Measurement of Gastric Emptying (GE)

In Experiments 1 and 2, the GE rate was evaluated using ultrasonography based on the change in cross-sectional area (CSA) of the gastric antrum before and after consumption of pumpkin soup. This approach provided a noninvasive method of GE assessment in real time. The method was previously confirmed to closely correlate with scintigraphy which is considered the gold standard for GE evaluation [20]. According to standard measuring method [20], cross-sectional image of gastric antrum was viewed with the left lobe of the liver, superior mesenteric vein, and abdominal aorta in a longitudinal section as reference markers using ultrasound sonography (Applio 300, Canon Medical Systems, Tokyo, Japan) with a 2.5-MHz convex probe. CSA of the maximal gastric antrum was determined by tracing the outer layer of the gastric wall (serosa) according to the method used in a previous study [21]. In accordance to the way of evaluating GE rate in a previous study [13], CSA of the gastric antrum immediately before (i.e., t = 0) and after (i.e., t = 15) consumption of the pumpkin soup was defined as 0% and 100%, respectively. The relative percent reduction in CSA at every time point was represented and calculated. CSA of the gastric antrum was measured at baseline, and 5-, 10-, and 20-min intervals for 15–20 (both experiments), 30–60 (both experiments), and 80–120 min (only Experiment 1) after consumption of pumpkin soup, respectively.

In Experiment 1, GE rate was also evaluated using the ^13^C-sodium acetate breath test [22]. This breath test is a widely-used, reproducible, and noninvasive measurement alternative to scintigraphy, devoid of radioactive exposure [22]. Next, 100 mg of ^13^C-sodium acetate was dissolved into pumpkin soup. Before and after consumption of pumpkin soup, breath samples were collected using capacity bag at 5-min intervals for 5–120 min and measured using an isotope ratio mass spectrometer (POCone; Otsuka Electronics, Hirakata, Japan). After experiment, GE rate index (i.e., Tmax-calc) was calculated according to a standard analysis method [22] and our previous study [23]. This parameter closely correlates with GE rate as measured by scintigraphic method [22,24,25] and the Wagner–Nelson method [26]. However, from the viewpoint of reducing the estimation error by curve fitting, the breath sample data for a long time are required (i.e., more than 60 min). Therefore, the mean values of Tmax-calc were over 60 min in Experiment 1, so ^13^C-sodium acetate breath test was not used in Experiment 2.

#### 2.4.3. Subjective Scores of Appetite and Food Intake

In Experiment 1, immediately after consumption of pumpkin soup, the participants reported their perceived food liking/disliking using Japanese translations of “Labeled Hedonic Scale” [23,27]. In Experiments 1 and 2, each subject’s motivation to eat was assessed by measuring desire to eat, hunger, fullness, and prospective consumption, using 100-mm visual analog scales using Japanese versions [23] before the start and at 5-, 10-, and 20-min intervals for 5–20 min (both experiments), 30–60 min (both experiments), and 80–120 min (only Experiment 1) from consumption of pumpkin soup, respectively. Using the scores of these four questions, a subjective average appetite score was calculated for each measurement time point according to the formula of the previous study [28].

Dry pasta was boiled in boiling water for eight minutes, and then the water was drained with a strainer. Next, the pasta was placed on a plate and well-mixed with the pasta sauce. The weight of the pasta was recorded before consumption using electronic scale (TE1502S; Sartorius) with a minimum reading of 0.01 g, and the energy intake per 1 g on a plate was calculated by using the manufacturer-reported values. Immediately after food intake, the total amount of food intake was recorded and energy intake was calculated.

### 2.5. Statistical Analysis

Data were presented as mean and standard deviation (SD) of the mean. In Experiment 1, the effects of time and oil on the time course of %GE rate were evaluated using two-way analysis of variance (ANOVA) for repeated measurements. If a significant main effect was detected, post-hoc Dunnett’s and paired *t*-test were conducted to determine the effects of time and oil (CO and HO), respectively. The effect of oil on the GE index using ^13^C breath test (i.e., Tmax-calc) and food liking were analyzed by paired t-test. In Experiment 2, the effects of time and oil on the time course of %GE rate and subjective appetite score were evaluated using two-way analysis of variance (ANOVA) for repeated measurements. If a significant main effect was detected, Dunnett’s and Tukey’s post-hoc tests were conducted to determine the effects of time and oil (CO, HO, and 2,4-DD), respectively. The effect of oil on food intake was evaluated using a one-way ANOVA for repeated measurements. If a significant main effect was detected, Tukey’s post-hoc tests were conducted. Statistical analyses were performed using SPSS version 18 (IBM Corp., Armonk, NY, USA). A *p*-value < 0.05 was considered significant.

## 3. Results

### 3.1. Subjective Food Liking Scores and 2,4-DD Concentrations

In Experiment 1, subjective food liking scores did not differ between CO and HO (31 ± 5 mm vs. 22 ± 6 mm, *p* > 0.05). In Experiment 1, concentrations of 2,4-DD, 2-octenal, and 2-heptenal in HO trial were 108 ppm, 9 ppm, and 36 ppm, respectively, while those in CO trial were below the detection limit (0.5 ppm, 0 ppm, and 0 ppm, respectively). In Experiment 2, concentrations of 2,4-DD in CO, HO, and 2,4-DD trials were 0 ppm, 327 ppm, and 346 ppm, respectively. Concentrations of 2-octenal and 2-heptenal in HO trial were 42 ppm and 228 ppm, respectively, while those in CO and 2,4-DD trials were not detected.

### 3.2. Gastric Emptying

**Figure 2:**
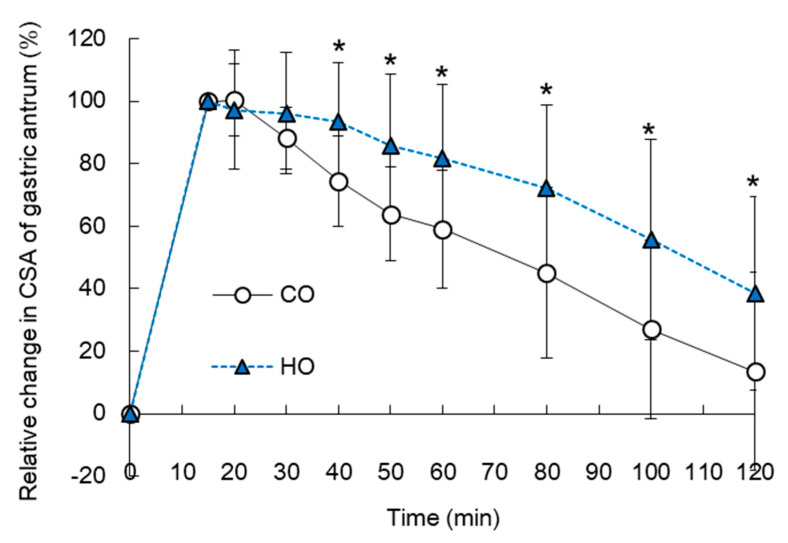
Time course of relative change in CSA of gastric antrum (i.e., % GE) following consumption of pumpkin soup containing 9.2 g of either intact corn oil (CO) or heated corn oil (HO) in Experiment 1. At 15 min after consumption of pumpkin soup, CSA of the gastric antrum was defined as 100%. Mean ± SD. * *p* < 0.05 (CO vs. HO).

**Figure 3:**
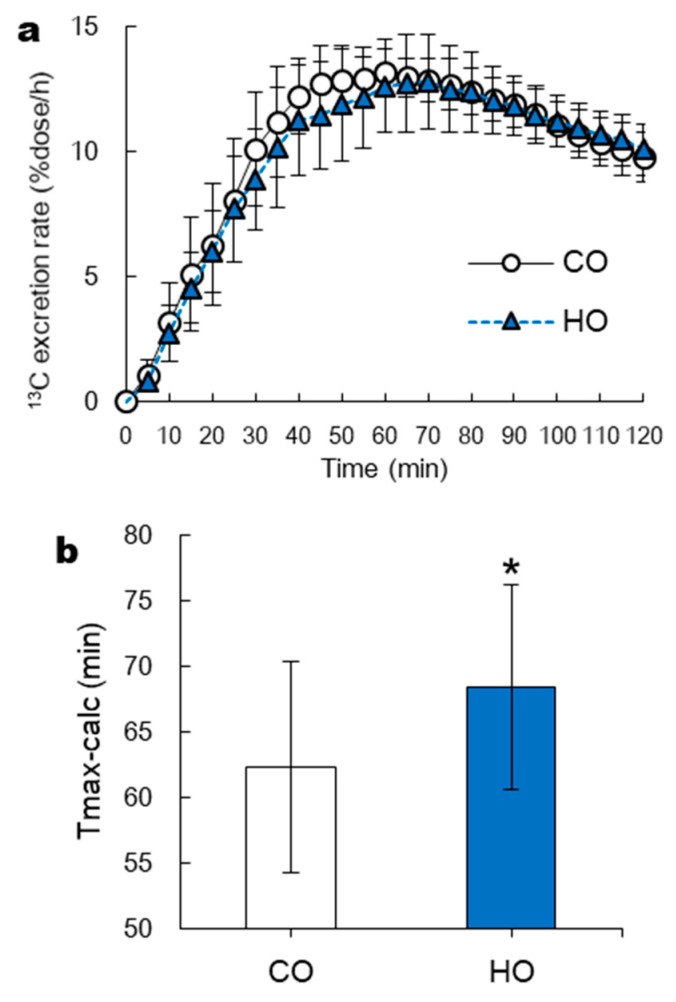
Time course of ^13^C excretion responses (**a**) and Tmax-calc of ^13^C excretion rate (**b**) following consumption of pumpkin soup containing 9.2 g of either intact corn oil (CO) or heated corn oil (HO) (**b**) in Experiment 1. Mean ± SD. * *p* < 0.05 (CO vs. HO).

In Experiment 1, two-way repeated-measures ANOVA showed a significant main effect of oil on the % GE. In Experiment 1, immediately before and 15 min after consumption of pumpkin soup, absolute CSA of the gastric antrum did not differ between CO and HO (immediately before consumption: 4.2 ± 0.8 cm^2^ vs. 4.2 ± 1.0 cm^2^; at 15 min after consumption: 10.6 ± 1.6 cm^2^ vs. 11.5 ± 0.5 cm^2^). At 40–120 min after consumption of pumpkin soup, % GE in HO trial was higher (i.e., the GE rate was slower) than in CO trial (Figure 2). The time course of the pulmonary [^13^CO_2_] excretion rate is shown in Figure 3a. Tmax-calc for HO trial was significantly delayed compared with CO trial (CO vs. HO, 62 ± 8 vs. 68 ± 8 min, *p* < 0.05) (Figure 3b).

**Figure 4:**
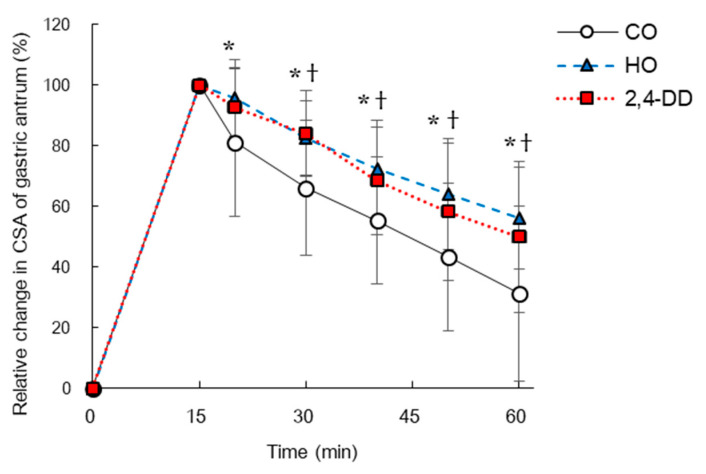
Time course of relative change in CSA of gastric antrum (i.e., % GE) following consumption of pumpkin soup containing 3.1 g of either intact corn oil (CO), heated corn oil (HO), or CO plus 1 mg of 2,4-decadienal (2,4-DD) in Experiment 2. At 15 min after consumption of pumpkin soup, CSA of the gastric antrum was defined as 100%. Mean ± SD. * *p* < 0.05 (CO vs. HO), † *p* < 0.05 (CO vs. 2,4-DD).

In Experiment 2, immediately before and 15 min after consumption of pumpkin soup, absolute CSA of the gastric antrum did not differ between the trials (immediately before consumption: 2.4 ± 1.2 cm^2^ in CO, 2.1 ± 1.1 cm^2^ in HO, and 2.5 ± 1.2 cm^2^ in 2,4-DD trial; 15 min after consumption: 9.0 ± 3.2 cm^2^ in CO, 8.4 ± 4.1 cm^2^ in HO, and 9.4 ± 3.1 cm^2^ in 2,4-DD trial, *p* > 0.05). At 20–60 min after consumption of pumpkin soup, % GE in HO and 2,4-DD (but not 20 min) trials were higher (i.e., the GE rate was slower) than in CO trials (*p* < 0.05) (Figure 4).

### 3.3. Subjective Scores of Appetite and Food Intake

In Experiments 1 and 2, two-way repeated-measures ANOVA did not show a significant main effect of oil on the ratings of desire to eat, hunger, fullness, prospective consumption, and total appetite score (*p* > 0.05) (Table 1 and Table 2). In both experiments, after consumption of pumpkin soup, the ratings of desire to eat, hunger, prospective consumption, and total appetite score slightly decreased from the baseline and then gradually returned to baseline values, whereas the ratings of fullness showed the opposite trend.

**Figure 5:**
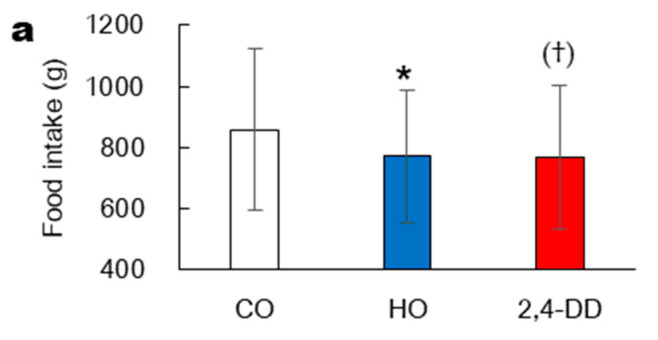

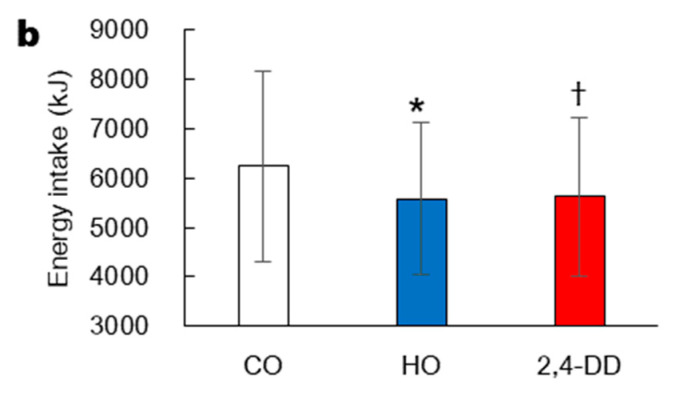
Food intake (**a**) and energy intake (**b**) at ad libitum test meal: 60 min after consuming pumpkin soup (240 g) containing 3.1 g of either intact corn oil (CO), heated corn oil (HO), or CO plus 1 mg of 2,4-decadienal (2,4-DD) in Experiment 2. Data are shown as mean ± SD. * *p* < 0.05 (CO vs. HO), † and (†) *p* < 0.05 and *p* = 0.06, respectively (CO vs. 2,4-DD).

In Experiment 2, one-way repeated-measures ANOVA showed a significant main effect of oil on total food intake and energy intake among the three trials. Food intake was lower in HO trial compared with CO trial (*p* < 0.05), while 2,4-DD trial had a tendency to lower food intake compared with CO trial (*p* = 0.06) (CO: 859 ± 265 g, HO: 773 ± 215 g, and 2,4-DD: 782 ± 229 g) (Figure 5a). The individual energy intake was shown (Supplement 1) and the mean values were lower in HO and 2,4-DD trials than in CO trial (*p* < 0.05) (CO: 6240 ± 1931 kJ, HO: 5586 ± 1529 kJ, and 2,4-DD: 5635 ± 1605 kJ) and that of HO trial did not differ from 2,4-DD trial and that of HO trial did not differ from 2,4-DD trial (Figure 5b).

## 4. Discussion

In Experiment 1, heated corn oil (HO trial) suppressed GE rate but did not affect subjective appetite scores. To our knowledge, two previous studies reported inconsistent effects of consumption of meal containing heated oil on GE rate in healthy humans. Namely, Benini et al. [13] reported that the ingestion of a fried meal with olive oil resulted in slower GE and longer persistence of gastric fullness compared with non-fried meal. However, Manning et al. [29] reported that ingestion of mash potato containing 50 g of heated sunflower oil did not affect GE rate in healthy humans. These differences might stem from different ways of measuring GE. Our study and Benini et al. [13] evaluated GE directly by measuring the time course of cross-sectional area of the gastric antrum using ultrasonography, whereas Manning et al. [29] used an indirect measurement method of GE (the paracetamol method). Orally ingested paracetamol is rapidly absorbed in the small intestine (but not in the stomach) and finally appears as blood paracetamol. However, our data on GE rate using ^13^C breath test (the method similar in principle to the paracetamol method) also showed significant differences between the HO and CO trials. Thus, other factors, such as nutrient content and composition of the meal, type of oil, and oil heating time may affect the GE rate irrespective of the way of measurement.

In Experiment 2, the amount of 2,4-DD produced by heating intact corn oil increased about three times compared with Experiment 1. In Experiment 2, HO trial suppressed GE rate even when the corn oil consumption was reduced from 9.2 g to 3.1 g. In addition, in the 2,4-DD trial (Experiment 2), GE rate was significantly suppressed compared with the CO trial, and it was equivalent to HO trial. This implies that 2,4-DD formed by oil heating is the main factor of GE suppression in HO trial. The reduced GE rate in the 2,4-DD trial may be partly explained by secretion of two kinds of gastrointestinal hormones. The first is CCK, which is produced from I-cells of the upper part of small intestine. In vitro (cell) studies showed the ability of 2,4-DD to enhance CCK secretion, which is involved in suppressing GE rate and increasing satiety [14]. Thus, the difference in GE rate between 2,4-DD and CO trials may stem from different CCK secretion, as discussed by Nakajima et al. [14]. The second relevant hormone is serotonin secreted from enterochromaffin cells, a type of endocrine cell present in the intestinal mucosal epithelium. Inhibitory effect of 2,4-DD on GE rate was shown in an in vivo study in rats, where it was found that 2,4-DD-containing diet inhibited GE rate by approximately 40% after ingestion compared with food without 2,4-DD [15]. Furthermore, Hira et al. [15] showed that pretreatment with tropisetron, a serotonin type 3 antagonist, in animals prevented the effects of 2,4-DD on GE rate. This suggests that 2,4-DD-induced suppression of GE rate is serotonin-mediated. Glucagon-like peptide-1 produced by K-cells, secretory cells in the upper part of the large intestine, is also a candidate as a regulator of GE rate and appetite [4,30]. However, a previous study in humans showed that glucagon-like peptide-1 responses did not differ between the heated and non-heated oil trials when sunflower oil was heated for 6 h at 180 °C and the oil and fat were mixed with mashed potato and ingested [29]. On the other hand, since no gastrointestinal hormones were measured in this study, it is difficult to clarify the mechanism of 2,4-DD-related inhibition of GE rate. Therefore, these gastrointestinal hormones (i.e., CCK, serotonin, and glucagon-like peptide-1) should be measured and evaluated simultaneously in a future study.

HO and 2,4-DD trials resulted in a lower energy intake than CO trial, corresponding to the results of GE rate. Reduced energy intake in 2,4-DD trial was almost equivalent to that in HO trial. This implies that consumption of 2,4-DD (either from heated oil or pure 2,4-DD added to intact oil) plays the key role in reducing food intake via suppression of GE rate. However, aldehydes other than 2,4-DD were formed in HO trial as Katragadda et al. [16] previously reported, which may act to induce CCK secretion [14]. For example, the HO trial contained 228 ppm of 2-heptenal and 41 ppm of 2-octenal. However, it has been reported that the effects of 2-heptenal, and 2-octenal on CCK secretion were relatively lower than the effects of 2,4-DD [14]. However, to explain the detailed mechanisms involved in GE rate and food control, further study is needed.

Our study had some limitations. In Experiment 2, food intake test was conducted at 60 min after consumption of pumpkin soup. This was based on the observation of suppression of GE rate and total appetite score at 60 min after consumption of pumpkin soup in Experiment 1. However, the eating manner in Experiment 2 does not mimic a general dietary situation where soup and spaghetti are simultaneously served. Thus, it is necessary to further examine the potential effects of heated oil and 2,4-DD on GE rate and food (energy) intake in general dietary situations. In Experiment 2, food (energy) intake was significantly lower for HO and 2,4-DD compared with CO trials. However, when the total sample size to determine food (energy) intake in this study was recalculated using G*Power software (version 3.1.9.2), an effect size of 0.721 for a critical F of 3.466 and power of 0.844 at α of 0.05 and power (1 − β) of 0.80 was obtained, and the calculated total sample size required was 24 participants. For that reason, our results should be interpreted with caution, and further work is necessary to better understand the effects of HO and 2,4-DD on food (energy) intake. In Experiment 2, we could not control menstrual cycles in female participants. A previous study reported that the menstrual cycle affects GE rate [17]. Although similar results were obtained in Experiments 1 and 2, we need to consider the menstrual cycle in order to obtain more precise results. In addition, the gender of the participants was female biased. However, Benini et al. [13] reported that GE rate after consumptions of either heated or non-heated meals with oil did not differ between men and women. On the other hand, Experiment 2 of this study is the first report on the effect of food energy intake on these oils and 2.4-DD. Therefore, to further generalize the results of this study, the ratio of male to female should be controlled equivalently, and the gender difference should be analyzed in the future studies. Moreover, we did not evaluate participants’ food liking/disliking of pumpkin soup in Experiment 2, considering that there was no difference in food liking between CO and HO trials in Experiment 1. Some previous studies demonstrated that unpleasant taste (bitter taste or unappetizing food) decreases gastric motility and GE rate [31,32]. However, participants sufficiently liked the food with HO in Experiment 1, and could not fully distinguish the differences of pumpkin soup with HO and 2,4-DD in Experiment 2. Therefore, food liking did not affect GE rate and subsequent food intake in our study.

## 5. Perspective and Conclusions

When the results of this study are applied to general dietary life, the following specific situations are assumed. For those who require dietary restriction, it can be suggested to add 2,4-DD-enriched oils to the meal or to use a cooking process in which 2,4-DD is formed. In the future, acute, short-term, and long-term interventional studies are needed that not only focus on healthy humans but also on obese individuals who need to manage overeating. In conclusion, 2,4-DD can suppress GE rate and energy intake in humans.

## Figures and Tables

**Table 1:** Participants’ appetite scores (mean values and standard deviations) in Experiment 1.

Time (min)	0	5	10	15	20	30	40	50	60	80	100	120
Desire to eat (mm)												
CO	56 ± 28	48 ± 13	50 ± 17	52 ± 18	56 ± 17	61 ± 15	64 ± 19	66 ± 22	68 ± 28	75 ± 30	76 ± 30	78 ± 31
HO	65 ± 19	45 ± 15	48 ± 17	49 ± 17	52 ± 17	55 ± 20	60 ± 16	62 ± 17	65 ± 16	71 ± 11	74 ± 13	82 ± 11
Hunger (mm)												
CO	67 ± 33	46 ± 14	49 ± 18	49 ± 18	54 ± 17	59 ± 16	65 ± 17	68 ± 18	69 ± 19	73 ± 24	76 ± 24	78 ± 28
HO	73 ± 14	46 ± 20	48 ± 19	50 ± 20	50 ± 22	55 ± 19	61 ± 17	63 ± 18	67 ± 17	73 ± 13	77 ± 13	84 ± 11
Fullness (mm)												
CO	21 ± 14	59 ± 18	53 ± 22	51 ± 23	49 ± 20	41 ± 20	35 ± 14	34 ± 14	29 ± 15	24 ± 14	20 ± 12	15 ± 14
HO	13 ± 12	48 ± 22	50 ± 22	51 ± 22	50 ± 24	44 ± 28	39 ± 21	37 ± 18	30 ± 16	23 ± 15	22 ± 15	17 ± 15
Prospective consumption (mm)												
CO	73 ± 17	51 ± 17	55 ± 18	56 ± 18	58 ± 17	62 ± 15	66 ± 14	67 ± 14	70 ± 16	73 ± 17	74 ± 18	77 ± 24
HO	77 ± 12	54 ± 19	56 ± 19	57 ± 20	55 ± 17	63 ± 21	60 ± 19	63 ± 19	65 ± 19	73 ± 15	74 ± 15	80 ± 15
Total appetite score (a.u.)												
CO	72 ± 16	44 ± 15	48 ± 20	50 ± 20	54 ± 18	60 ± 17	66 ± 14	68 ± 14	71 ± 16	76 ± 16	78 ± 15	82 ± 15
HO	78 ± 10	48 ± 18	49 ± 18	50 ± 19	52 ± 19	58 ± 20	60 ± 17	62 ± 17	66 ± 17	74 ± 12	76 ± 13	83 ± 10

CO and HO mean intact corn oil and heated corn oil, respectively.

**Table 2:** Participants’ appetite scores (mean values and standard deviations) in Experiment 2.

Time (min)	0	5	10	15	20	30	40	50	60
Desire to eat (mm)									
CO	62 ± 29	52 ± 26	50 ± 24	55 ± 24	55 ± 25	57 ± 24	57 ± 23	60 ± 23	62 ± 24
HO	65 ± 24	52 ± 20	54 ± 22	57 ± 27	60 ± 27	63 ± 28	61 ± 28	63 ± 28	64 ± 27
2,4-DD	61 ± 26	51 ± 20	54 ± 21	56 ± 21	59 ± 21	58 ± 22	61 ± 26	61 ± 27	62 ± 26
Hunger (mm)									
CO	64 ± 29	47 ± 28	52 ± 26	54 ± 25	53 ± 25	53 ± 23	57 ± 26	61 ± 26	65 ± 25
HO	63 ± 29	48 ± 24	52 ± 23	56 ± 29	57 ± 27	57 ± 29	59 ± 29	58 ± 29	60 ± 29
2,4-DD	63 ± 26	44 ± 21	49 ± 24	52 ± 20	55 ± 23	57 ± 23	59 ± 25	57 ± 28	63 ± 26
Fullness (mm)									
CO	25 ± 30	39 ± 22	43 ± 25	43 ± 23	42 ± 24	45 ± 23	37 ± 27	33 ± 24	33 ± 25
HO	27 ± 26	42 ± 22	45 ± 26	45 ± 29	42 ± 28	40 ± 28	38 ± 28	37 ± 27	37 ± 29
2,4-DD	26 ± 26	42 ± 24	47 ± 25	45 ± 24	41 ± 24	39 ± 25	43 ± 28	41 ± 29	38 ± 29
Prospective consumption (mm)									
CO	74 ± 22	63 ± 22	66 ± 22	64 ± 19	65 ± 20	66 ± 20	67 ± 21	67 ± 21	69 ± 22
HO	68 ± 25	55 ± 23	57 ± 24	60 ± 28	63 ± 29	62 ± 28	64 ± 27	64 ± 28	65 ± 26
2,4-DD	71 ± 24	58 ± 19	58 ± 22	61 ± 25	63 ± 23	61 ± 23	61 ± 25	65 ± 25	65 ± 26
Total appetite score (a.u.)									
CO	69 ± 25	56 ± 22	56 ± 22	58 ± 21	58 ± 22	58 ± 20	61 ± 23	64 ± 21	66 ± 22
HO	67 ± 25	53 ± 21	55 ± 2	56 ± 26	59 ± 27	60 ± 27	61 ± 27	62 ± 28	63 ± 27
2,4-DD	67 ± 24	53 ± 18	54 ± 21	56 ± 21	59 ± 21	59 ± 22	60 ± 24	60 ± 24	63 ± 25

CO, HO, and 2,4-DD mean intact corn oil, heated corn oil, and CO plus 1 mg of 2,4-decadienal, respectively.

## Data Availability

Data available on request due to restrictions e.g., privacy or ethical from the corresponding author.

## Supplementary Materials

The following are available online at https://www.mdpi.com/article/10.3390/nu13041304/s1, Table S1: The individual energy intake at ad libitum test meal in the Experiment 2.
