# Supplementary material for: Heated Corn Oil and 2,4-Decadienal Suppress Gastric Emptying and Energy Intake in Humans
Source: Nutrients. 2021 Apr 15;13(4):1304. doi: 10.3390/nu13041304 (PMC8071276; doi:10.3390/nu13041304)
Supplement: Supplementary file 1 [file nutrients-13-01304-s001.zip › nutrients-1128407-supplementary.pdf]

Supplement 1

The individual energy intake at ad libitum test meal in the Experiment 2

Participant number	CO (kJ)	HO (kJ)	2,4-DD (kJ)
1	5867	6046	5573
2	8216	6282	7950
3	4903	3582	3715
4	6084	4420	5402
5	2761	3275	3507
6	7886	7431	6905
7	4673	5573	5573
8	6553	5573	5573
9	5573	4915	5573
10	9288	7431	9288
11	8889	7690	6174
12	7431	6606	4732
13	3112	2837	2988
14	4530	4754	4572
15	7431	7431	7431
16	7872	6455	5573
17	5015	4657	5271
Mean	6240	5586	5635
SD	1931	1529	1605

CO, HO and 2,4-DD mean intact corn oil, heated corn oil, and CO plus 1 mg of 2,4-decadienal, respectively.
